# Increasing prevalence of ESBL production among Irish clinical Enterobacteriaceae from 2004 to 2008: an observational study

**DOI:** 10.1186/1471-2334-12-116

**Published:** 2012-05-15

**Authors:** Jérôme Fennell, Akke Vellinga, Belinda Hanahoe, Dearbhaile Morris, Fiona Boyle, Francis Higgins, Maura Lyons, Karina O’Connell, Deirbhile Keady, Martin Cormican

**Affiliations:** 1 Department of Medical Microbiology, Galway University Hospitals, Galway, Ireland; 2 Discipline of General Practice, School of Medicine, NUI Galway, Ireland; 3 Discipline of Bacteriology, School of Medicine, NUI Galway, Ireland

## Abstract

**Background:**

Extended spectrum β-lactamase (ESBL) producing *Enterobacteriaceae* infections are associated with delayed initiation of appropriate treatment, poor outcomes and increased hospital stay and expense. Although initially associated with healthcare settings, more recent international reports have shown increasing isolation of ESBLs in the community. Both hospital and community ESBL epidemiology in Ireland are poorly defined.

**Methods:**

This report describes clinical and laboratory data from three hospitals over 4.5 years. All significant isolates of *Enterobacteriaceae* were subjected to standardized antimicrobial susceptibility testing and screening for ESBL production. Available patient data from hospital databases were reviewed.

**Results:**

The database included 974 ESBL producing organisms from 464 patients. Urine and blood isolates represented 84% and 3% of isolates respectively. *E. coli* predominated (90.9%) followed by *K. pneumoniae* (5.6%). The majority of patients (n = 246, 53.0%) had been admitted to at least one of the study hospitals in the year prior to first isolation of ESBL. The overall 30-day all-cause mortality from the date of culture positivity was 9.7% and the 1 year mortality was 61.4%. A Cox regression analysis showed age over 60, male gender and previous hospital admissions were significant risk factors for death within 30 days of ESBL isolation. Numbers of ESBL-producing *E. coli* isolated from urine and blood cultures increased during the study. Urine isolates were more susceptible than blood isolates. Co-resistance to other classes of antimicrobial agents was more common in ESBL producers from residents of long stay facilities (LSF) compared with hospital inpatients who lived at home.

**Conclusions:**

This work demonstrates a progressively increasing prevalence of ESBL *Enterobacteriaceae* in hospital, LSF and community specimens in a defined catchment area over a long time period . These results will improve clinician awareness of this problem and guide the development of empiric antimicrobial regimens for community acquired bloodstream and urinary tract infections.

## Background

Since the first report of a plasmid-encoded extended spectrum β-lactamase (ESBL) producing *Enterobacteriaceae* in 1983 [1], ESBLs have continued to increase in variety and prevalence and are now a global health concern. The spread of ESBLs has implications for clinicians and patients as ESBLs have been associated with delays in effective treatment [2], poor outcomes [3,4] increases in hospital stay [5] and health care costs [6,7]. ESBLs are commonly resistant to other antimicrobial agents because of mobile genetic elements encoding other antimicrobial resistance determinants and/or chromosomal mutations. The co-resistance to other agents reduces the antimicrobial treatment options available and may enable selection for ESBLs by non-beta-lactam antimicrobials such as the aminoglycosides and fluoroquinolones.

While traditionally associated with healthcare settings, more recent reports have shown increasing isolation of ESBLs in the community setting [8]. Nursing homes and other long stay facilities have been proposed as a reservoir for ESBLs in the community [9-11]. The residents often have risk factors for ESBL acquisition such as antimicrobial exposure, prior hospital admission, incontinence, urinary catheters and decubitus ulcers. In the UK and Ireland, CTX-M enzyme producing organisms in the community have emerged as a serious problem [12] and have been associated with increasing resistance rates to other antimicrobial agents [13].

The first report of an ESBL in Ireland was of an outbreak of *Klebsiella pneumoniae* in a paediatric hospital in 1998 [14]. Subsequent assessments of ESBL epidemiology in Ireland have been limited to small numbers of isolates. The most comprehensive data on ESBL prevalence in Ireland to date is from the European Antimicrobial Resistance Surveillance System (EARSS). This surveillance, which is limited to blood culture isolates shows an increase in the prevalence of ESBL amongst *E. coli* from 1.2% in 2002 to 5% in 2008 [15]. With respect to isolates from other sites NíChulain et al. [16] reported a prevalence of 0.3% amongst *E. coli* from urine samples (hospital and community) from the West of Ireland in the period 2002–2003. Two of these ESBL isolates were from the community. Cefotaximase-producing (CTX-M) *E. coli* were first reported from Ireland in 2005 [17] and were associated with a long stay facility (LSF) outbreak soon afterwards in 2006 [18]. To date there has been no comprehensive longitudinal report of ESBL prevalence from all clinical specimen types from Ireland.

The objective of this report is to describe the changing prevalence of ESBLs from all clinical sites in the hospital and community over a period of 4.5 years. The data clarify the extent of dissemination of ESBLs in the hospital and community and should inform clinical and public health awareness of this growing problem.

## Methods

### *Setting*

Galway University Hospitals (GUH: University College Hospital Galway (UCHG) and Merlin Park Hospital) and Roscommon County Hospital have a combined total of 1052 inpatient beds and about 44,000 admissions per year. They are the major hospital service providers for counties Roscommon and Galway in the west of Ireland. The Clinical Microbiology service at GUH serves these hospitals, approximately 90 nursing homes and General Practitioners in much of Galway, Roscommon and parts of Mayo. The population of these 3 counties in 2006 was 341,863 (http://www.cso.ie).

### *Bacterial isolates*

All hospital and community isolates of *Enterobacteriaceae* cultured from clinical samples between July 2004 and December 2008 were screened for ESBL production. Isolates from surface swabs, stool samples and rectal swabs were excluded. Organisms were identified based on colonial morphology on Chromagar and confirmed with the VITEK-2® (bioMérieux, France). Throughout the period routine susceptibility testing was performed by the disc diffusion method of the Clinical Laboratory Standards Institute (CLSI, formerly NCCLS) with supplementary determination of MIC with Etest [19,20]. Routine quality control was performed with appropriate strains as specified in CLSI documents. All significant urine isolates (>100,000 CFU/ml pure culture) of *Enterobaceriaceae* that were resistant to ampicillin were also tested for susceptibility to coamoxiclav, ciprofloxacin, trimethoprim, nitrofurantoin, cephalothin, cefuroxime, cefotaxime, ceftazidime, piperacillin/tazobactam, nalidixic acid, ofloxacin, nalidixic acid, gentamicin, and amikacin. All significant *Enterobacteriaceae* from sites other than urine were tested for susceptibility to ampicillin, coamoxiclav, cephalothin, cefuroxime, cefotaxime, ceftazidime, piperacillin/tazobactam, meropenem, nalidixic acid, ciprofloxacin, gentamicin and amikacin.

From July 2004 to end of March 2005 ESBLs were identified based on the then current NCCLS criteria for screening for ESBLs with cefotaxime (zone diameter ≤27 mm) and cefuroxime (zone diameter ≤22 mm). From April 2005 to December 2008 first line susceptibility testing of all urinary *Enterobacteriaceae* was expanded to include cefpodoxime to increase sensitivity of ESBL detection. Suspect ESBLs were evaluated with cefpodoxime and cefpodoxime/clavulanate discs (Mast UK) by CLSI criteria confirmed with ESBL Etests® strips according to the manufacturer’s instructions (AB Biodisk, Solna, Sweden).

### *Patient data*

Galway University Hospitals and Roscommon County Hospital share a networked patient and laboratory information system. Data on all ESBL isolates were extracted and manually cross-referenced between the laboratory and hospital databases. For each patient the request date, date of admission, time from admission to culture, date of discharge, 30 day mortality, 1 year mortality, date of death, admission to any of the three hospitals in the previous year, hospital number, specimen number, specimen type, patient name, date of birth, clinician name, location, hospital code, patient address and age were recorded. Permission for the study was granted by the Director of Pathology. Ethical permission was granted by the head of the hospital ethics committee.

### *Statistical analyses*

For all analysis except the comparison with the EARSS data, only the first isolate was included for each patient. To compare the prevalence of resistance between community patients (i.e. patients not in hospital or a LSF at time of isolation), hospitalised inpatients and LSF residents, a *X*^2^ for trend was performed. For analyses over time and for purposes of comparison with EARSS data, quarterly results were calculated in which only one isolate per patient per quarter was counted. The results were plotted and a simple linear regression line was calculated. Survival times were calculated from the date of isolation of the ESBL by the laboratory to the date of death. Patients still alive at the end of the study or of whom the outcome was uncertain were censored and the survival time was calculated based on the final date of the study. A Cox regression analysis using backward conditional regression was done using the number of days between the detection of the ESBL and either death or survival up to the first of June 2009 was counted. The model produces a survival function that predicts the probability of death at a given time t for given values of the predictor variables. Predictor variables entered in the model were age, residence in a nursing home, *E.coli* or other isolate, gender and admission to hospital in the year previous to the detection of the ESBL. The resulting hazard ratios and their 95% confidence interval can be compared with (and interpreted as) an odds ratio obtained from a logistic regression. The hazard ratio obtained from the Cox regression analysis adjusts each variable for the confounding effect of the others. Interaction between the variables was checked but not found to be significant. Two models were checked, the first assuming all patients of whom no further records were found, were still alive at the end of the study period, the second one excluding the patients of whom no certainty was obtained concerning their status. Both models showed the same outcomes and only the first is presented here. All analyses were done with SPSS for Windows version 18.0. The p-value for significance was set at 0.05.

## Results

Over the 4.5 year period 974 ESBL isolates were cultured from all sites from 464 patients. Of these patients 304 (65.5%) were female, 391 (84.3%) isolates were from urine, 29 (6.3%) sputum and 16 (3.5%) from blood cultures. Considering the first isolate per patient (n = 464) *E. coli* was the predominant species (422, 91.0%) followed by *Klebsiella pneumoniae* (26, 5.6%), Enterobacter species (10, 2.2%) and the remaining isolates consisting of *Morganella morganii* (3, 0.6%), *Citrobacter freundii* (2, 0.4%) and *Proteus mirabilis* (1, 0.2%). The majority of patients (n = 246, 53.0%) had been admitted to at least one of the study hospitals in the year prior to first isolation of ESBL. The overall 30-day all-cause mortality from the date of culture positivity was 9.7% and the 1 year mortality was 61.4%.

A slight majority of samples were from hospital inpatients (256, 55.4%) with LSF residents accounting for 26.2% (n = 67) of the hospital inpatients. Most of the hospital inpatients who were residents of LSF (n = 54) has been admitted to one of the study hospitals in the previous year. Of the 233 hospital patients with admission data available, ESBLs were cultured from 66 (28.3%) within 2 days of admission. Of the patients who cultured an ESBL more than 2 days after admission, the median period from admission to ESBL culture was 16 days (3–801). Just under half patients (44.6%) were from the community with LSF residents representing 34.5% of the 206 community patients. It is important to note however 89 (43.2%) of the community patients were not admitted to hospital in the previous year and did not reside in a LSF.

At the beginning of April 2005 (end of third quarter) the approach to screening for ESBL was changed from the application of NCCLS criteria for ceftazidime and cefotaxime to ampicillin-resistant *Enterobacteriaceae* to universal testing for susceptibility to cefpodoxime using the CLSI method. The number of ESBLs detected immediately increased from 17 isolates (from all sites) in the first 9 months before the change in method to 61 in the next 9 month period. This step change in the second quarter of 2005 is therefore related to a change in method however the screening approach then remained constant and the subsequent pattern is one of a progressive increase in the number of ESBLs isolated per quarter to a peak of 73 in the last quarter of 2008.

**Figure 1:**
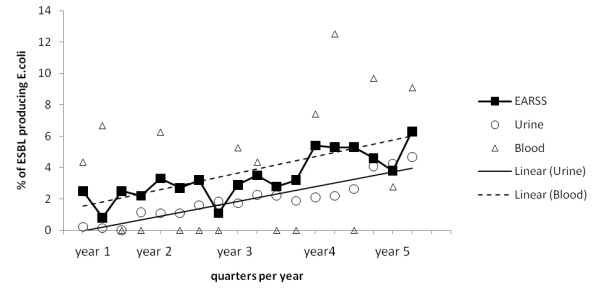
**ESBL* E. coli *as a percentage of* E. coli *isolated from blood and urine per quarter (July 2004 – December 2008).** Horizontal axis time line begins July 2004. Triangles represent % ESBL *E. coli* from blood in the study hospitals and circles represent the % ESBL *E. coli* from urine per quarter. The dashed line and solid lines represent the respective linear trends for blood and urine. Solid black squares show national data for ESBL *E. coli* from blood cultures per quarter from the European Antimicrobial Resistance Surveillance System.

The percentage of ESBL *E.coli* isolated from urine and blood per quarter were calculated to compare to national EARSS data (Figure 1). Duplicate samples were removed from both the numerator and denominator groups on a quarterly basis. *E. coli* ESBL bacteremias increased from 1.6% of isolates in the first year of CLSI ESBL detection (April 2005 to March 2006) to 5.4% in the final 12 months of ESBL detection (January to December 2008). The national EARSS data showed an increase from 2.8% to 5% for the corresponding time periods. ESBL *E. coli* isolates from urine increased from 1.2% to 3.9% for the corresponding 12 months periods.

The quarterly rate for ESBL *E. coli* in urine and blood cultures was compared to national EARSS data. The mean prevalence of ESBL-producing *E. coli* from bacteremias was 3.8% which corresponded closely with the EARSS rate of 3.4%. The linear regression was significant for urine (R^2^ 87.4%, p <0.001) but not for the blood cultures (R^2^ 11.6%, p > 0.1). This may be related to the relatively low numbers of blood culture isolates.

The resistance of blood and urinary ESBL producing *E. coli* to other antimicrobial agents was assessed (Table 1). All blood culture isolates were resistant to ciprofloxacin and susceptible to meropenem. Nitrofurantoin was the most consistently active non-betalactam agent against ESBL isolates from urine. Changes in % resistance over time were assessed for urine isolates. The % resistance to nitrofurantoin changed very little (1.2% increase) over the period studied. Other oral agents showed a greater increase in resistance, with % resistance to ciprofloxacin and trimethoprim increasing by 14.4% and 29.3% respectively. The % resistance to amikacin showed a 5.2% increase and to gentamicin a 7.8% decrease.

**Table 1:** **Antimicrobial Resistance in ESBL-producing* E. coli *from Blood (N = 15) and Urinary (N = 366)**

	**Susceptible (%)**		**Intermediate (%)**		**Resistant (%)**	
	**Blood**	**Urine**	**Blood**	**Urine**	**Blood**	**Urine**
Amikacin	11 (78.6)	312 (86.4)	2 (14)	29 (8.0)	1 (7.1)	20 (5.5)
Gentamicin	9 (60.0)	254 (70.2)	1 (7)	3 (0.8)	5 (33.3)	105 (29.0)
Ciprofloxacin	0 (0)	62 (17.0)	0 (0)	1 (0.3)	15 (100)	302 (82.7)
Meropenem	15 (100)	-	0 (0)	-	0 (0)	-
Trimethoprim	-	68 (18.6)	-	0 (0)	-	297 (81.4)
Nalidixic Acid	-	43 (11.8)	-	1 (0.3)	-	319 (87.9)
Nitrofurantoin	-	323 (88.5)	-	20 (5.5)	-	22 (6.0)

Legend to Table 1. Susceptibility, Intermediate and Resistance to non-beta-lactam antimicrobial agents by Clinical Laboratory Standards Institute criteria for ESBL producing *E. coli* for the period July 2004 to December 2008. Note: one or more isolates had incomplete susceptibility data therefore the totals for each row do not fully correspond.

Urine ESBL-producing *E. coli* isolates were divided based on three patient categories, (1) those resident in the community (i.e. not in hospital or LSF) at time of isolation, (2) hospitalised patients who did not normally reside in a LSF and (3) residents of a LSF whether in hospital or in the LSF at the time of isolation of ESBL. The merging of hospitalised and non hospitalised LSF patients was based on the absence of significant differences between the data when the groups were considered separately. As shown in Table 2, the prevalence of antimicrobial co-resistance to non-betalactams in urine ESBL *E. coli* shows a significantly increasing trend in the prevalence of co-resistance from community to hospital to LSF patients for all antimicrobials except for nitrofurantoin.

**Table 2:** **Antimicrobial resistance in ESBL* E. coli *from urine by residence and hospitalization status**

	**Community**	**Hospital inpatients**	**LSF**	**Total (% resistant)**	**P value for trend**
Amikacin, n (%)	4 (20.0)	5 (25.0)	11 (55.0)	20 (6.0)	0.033
Gentamicin, n(%)	26 (24.8)	34 (32.4)	45 (42.9)	105 (29.2)	0.002
Ciprofloxacin, n (%)	89 (29.5)	101 (33.4)	112 (21.9)	302 (83.0)	0.000
Trimethoprim, n (%)	98 (33.0)	93 (31.3)	61 (35.7)	297 (81.4)	0.006
Nalidixic Acid, n (%)	102 (32.0)	104 (32.6)	113 (35.4)	319 (88.1)	0.000
Nitrofurantoin, n (%)	8 (36.4)	4 (18.2)	10 (45.5)	22 (6.4)	n.s.

Legend to Table 2. The prevalence of ESBL *E. coli* (%) resistant to non beta-lactam antimicrobial agents by patient status. Community means those resident in the community at time of isolation, hospitalised means hospitalised patients who normally reside in the community and LSF refers to residents of a LSF whether in hospital or in the LSF at the time of isolation of ESBL *E. coli*. P values indicate the significance of a trend of increasing prevalence of resistance from the community population through to the patient population resident in an LSF. Note comparison of data for hospitalised and non-hospitalised LSF patients showed no significant difference.

Data for a survival analysis were available for 464 patients of whom 124 had a known date of death and the others for whom the survival time was censored at the final study date. Overall the median survival time was 488 days (from 0–1856 days). The median survival time of the deceased patients was 76 days (0–926 days). The 30 day all-cause mortality was 9.7%. A Cox regression analysis of the survival times of patients with an ESBL infection was performed. Included risk factors were age, gender, nursing home resident, *E.coli* ESBL and a hospital admission in the year prior to the ESBL isolation. The interpretation of the hazard ratio and 95% Confidence Interval (CI) is similar to that for the odds ratio. Hazard ratios of 1 mean the groups do not differ in the time to death, a hazard ratio > 1 means that the group of interest has a shorter time to death compared to the reference group (i.e. this group is at ‘higher risk’ of death). Only factors with a p value of less than 0.05 were kept in the model. No interactions were significant.

**Figure 2:**
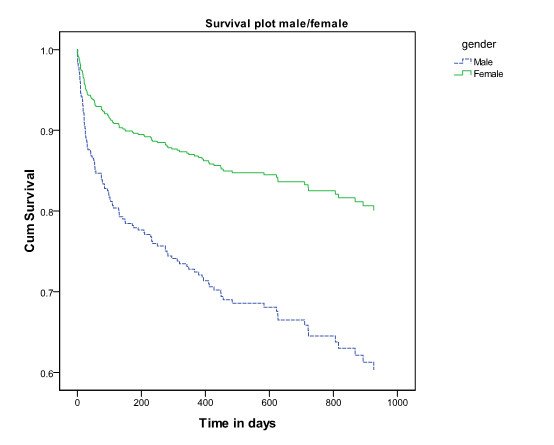
**Survival plot by gender of patients from whom ESBLs were isolated.** Legend to Figure 2. Cumulative survival of patients from whom ESBLs were isolated by gender. The upper (green) line is female, the lower blue line is male.

**Figure 3:**
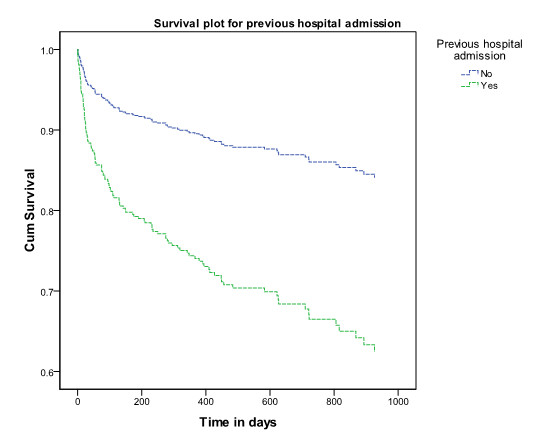
**Survival plot by history of hospitalisation in the year prior to isolation of ESBL.** Legend to Figure 3. Cumulative survival of patients from whom ESBLs were isolated by history or hospital admission in the year prior to isolation of ESBL. The upper (blue) represents survival of those without history of admission, the lower (green) represents survival of those with a history or hospitalisation.

**Figure 4:**
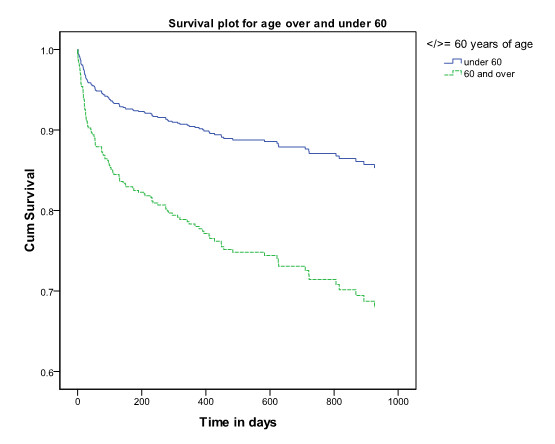
**Survival plot for patients from whom ESBLs were isolated by age over or under 60 years of age.** Legend to Figure 4. Cumulative survival of patients from whom ESBLs were isolated by age over and under 60 years. The upper (blue) line represents survival of those under 60, the lower (green) line represents survival of those over 60.

Age, gender and previous hospital admission were significant risk factors in the model. Being admitted to hospital in the year prior to the isolation of the ESBL increased the hazard ratio to 2.7 (95% CI 1.8-4.2). Being male increased the hazard ratio to 2.3 (95% CI 1.6-3.3) and for every year older the hazard ratio increased with 1.03 (1.01-1.04). When age was categorised into those over and under 60, the hazard ratio was 2.4 (95% CI 1.4-4.3) for the older group. Figures 2,3 and 4 show the survival plots for gender, previous hospitalisation and age above and below 60.

## Discussion

This work examined 4.5 years of clinical epidemiological and laboratory data of ESBL cultures from all clinical sites from 3 Irish hospitals. This is the first extensive investigation of the epidemiology of ESBL producing *Enterobacteriaceae* from all specimen types in Ireland. The broad trends are similar to that observed in other countries. The predominance of female patients (65%) and urine samples (84%) is consistent with previously published studies[21-23]. The similar numbers of isolates from the hospital setting (54%) and the community setting (46%) confirms the extent to which ESBLs have extended beyond the confines of the hospital into the wider community. It is particularly important to note that a high proportion (42%) of community isolates are from people who do not live in nursing homes and had no history of hospital admission in the previous year and who therefore may not be identified as at risk for infection with antimicrobial resistant *Enterobacteriaceae*. However it is a limitation of the study that some of these patients may have been hospitalised elsewhere during the previous year. This finding is however consistent with a Spanish study which showed that almost half of the community patients had no history of hospital admission in the previous year and had not been in a LSF [23].

From a clinical perspective the data indicate that at present meropenem remains a reliable agent for treatment of ESBLs and amikacin is also active in most cases. Nitrofurantoin is important in that it is active against most ESBLs and there is no clear trend to increasing resistance. We have not included data on beta-lactams other than meropenem in the analysis however it is appropriate to note that a significant number of isolates are susceptible in vitro to coamoxiclav and piperacillin/tazobactam. The role of these agents in therapy of ESBL related infection has been the subject of much debate although recently a consensus has emerged that with the application of current interpretive criteria for beta-lactams agents an in-vitro susceptible result may be accepted clinically relevant (http://www.eucast.org).

The change in detection method from the application of cefotaxime/ceftazidime screening criteria to ampicillin-resistant isolates to the screening of all isolates with cefpodoxime in April 2004 resulted in a dramatic step increase in the number of ESBLs detected. In Ireland as in other countries, the extent of ESBL dissemination has most likely been underestimated due to limited application of appropriate detection methods in Irish laboratories as has been shown in other countries [24]. Correct determination of the mechanism of beta-lactam resistance may be less important in the future for clinical purposes as newer, lower breakpoint criteria from CLSI and EUCAST are likely to be more effective at differentiating between those isolates that are likely to respond to therapy and those which will not. Nevertheless detection of the mechanism remains important for epidemiological and infection control purposes and this paper underlines the importance of comprehensive testing for novel resistance mechanisms in isolates from a broad range of specimen types from hospital and community may have particular relevance to monitoring the current emergence of carbapenemase producing *Enterobacteriaceae*.

Our assessment shows an increase in the percentage of ESBL-producing *E. coli* from both blood and urine isolates over the time period studied and this had reached 9.0% and 4.7% respectively by the end of the study. In 2009 this trend continued and 5% of urine *E. coli* isolates and 11.9% of blood culture *E. coli* isolates were ESBL producers. These trends closely parallel the results seen in the Irish EARSS data. This indicates that the EARSS data are a useful barometer of antimicrobial resistance trends in clinical *E. coli* isolates other than blood. The principle limitation of EARSS data is that by the nature of blood stream infection it tends to focus on those hospitalised with serious illness and therefore is not sensitive to trends in the community.

Residence in a nursing home is widely recognised as a risk factor for acquisition for ESBLs and we and others have published evidence of transfer of isolates between nursing home residents [18,25]. However the finding that ESBL isolates from residents of nursing homes are associated with higher levels of co-resistance to other classes of antimicrobial agents, in particular higher levels of co-resistance than observed in ESBL isolates from hospital patients (other than those admitted from nursing homes) was unexpected. This suggests that nursing homes may play an important part in amplification and dissemination of multiply-resistant ESBLs. Characterisation of ESBLs to determine relationships at a molecular level and define the specific genetic basis of resistance was not undertaken in this study.

### *Mortality*

The data indicate that in patients from whom ESBLs have been isolated overall 30 day all-cause mortality was 9.7% and that male sex, increasing age and previous hospital admission were independently associated with higher mortality. The same risk factors were shown to be associated with community ESBL infections by Ben-Ami et al. [26]. The clear association of age with mortality would be expected in the light of increasing morbidity with age. Urinary tract infections in males are generally less common and associated with anatomical abnormalities and therefore males in this study may represent a cohort of individuals with significant comorbidity related to prostatic obstruction and other factors. This may explain the increased mortality of males compared to females.

This study has a number of limitations. There was limited clinical data, no data on antimicrobial exposure and no control group. Details of co-morbidities and risk factors such as diabetes mellitus, liver disease, malignancy, renal dysfunction, presence of long term urinary catheters or inadequate empirical therapy are absent. As we did not have access to all databases of all hospitals it is possible that some patients were misclassified with respect to hospital admission in the previous year. Nevertheless we believe the data contribute to improved understanding of the epidemiology of ESBL transmission and in particular the extent to which ESBLs are now common even in those with no apparent prior engagement with the health care services. Reducing and optimising antimicrobial use across all sectors is accepted as a key intervention to limit the spread of acquired antimicrobial-resistance. A 2008 report examining antimicrobial consumption in Irish Acute hospitals (http://www.hpsc.ie/hpsc/A-Z/MicrobiologyAntimicrobialResistance/EuropeanSurveillanceofAntimicrobialConsumptionESAC/SurveillanceReports/File,3889,en.pdf) demonstrated relatively high levels of antimicrobial consumption in Irish hospitals. Community antibiotic prescribing data was not readily available but Ireland was reported as one of only 3 European countries where community prescriptions were increasing[27]. There is clearly much work to be done to improve standards of antimicrobial stewardship.

The particular association of high levels of co-resistance to other classes of antimicrobial agents with isolates from residents of nursing homes is of concern. The extent to which it is possible to limit spread of antimicrobial-resistant Enterobacteriaceae in nursing homes is questionable. It is generally not practical in a nursing home to achieve the standards of infection control practice that are expected in an acute care setting. It would almost certainly not be possible to maintain the extent of patient segregation that is common in acute hospitals. Furthermore antimicrobial consumption in nursing homes is likely to be difficult to restrict. Supporting older people to continue to live in their own communities may be the most effective approach to addressing this problem in addition to the many other benefits associated with maintaining independent living for as long as possible.

## Conclusions

This study was the first extensive epidemiological assessment of Irish ESBL-producing isolates and reveals their increasing prevalence in community, hospital and long stay patients and gives a preliminary assessment of the associated mortality. The finding that ESBL isolates from residents of nursing homes are associated with higher levels of co-resistance to other classes of antimicrobial agents, in particular higher levels of co-resistance than isolates from hospital patients not admitted from nursing homes was unexpected and worrying. This suggests that nursing homes may play an important part in amplification and dissemination of multiply-resistant ESBLs. The data should help improve clinical and public health awareness of this issue and aid development of local empiric antimicrobial regimens for community acquired bloodstream and urinary tract infections.

## Competing interests

All authors declare no competing interests.

## Authors’ contributions

JF designed the study, collected the data and wrote the manuscript. AV aided in the study design, performed the entire statistical analyses, reviewed and contributed to the manuscript. BH and KOC helped collect the data, review and revise the manuscript. DM, FB, FH and ML conducted much of the laboratory testing and reviewed and contributed to the manuscript. MC oversaw the entire project and both MC and DK aided in the study design, reviewed and helped draft the manuscript. All authors read and approved the final manuscript.

## Pre-publication history

The pre-publication history for this paper can be accessed here:

http://www.biomedcentral.com/1471-2334/12/116/prepub
